# Supplementary material for: Cyclopamine modulates smoothened receptor activity in a binding position dependent manner
Source: Commun Biol. 2024 Sep 28;7:1207. doi: 10.1038/s42003-024-06906-y (PMC11438977; doi:10.1038/s42003-024-06906-y)
Supplement: Supplementary file 1 — Supplementary Material [file 42003_2024_6906_MOESM1_ESM.pdf]

Supplementary Information

Cyclopamine modulates Smoothened Receptor Activity in a Binding Position Dependent Manner

Kihong Kim,[†] Prateek Bansal,[†] and Diwakar Shukla[*,†,‡,¶,§]

†*Department of Chemical and Biomolecular Engineering, University of Illinois at Urbana-Champaign, Urbana, IL, 61801, United States*

‡*Department of Bioengineering, University of Illinois at Urbana-Champaign, Urbana, IL, 61801, United States*

¶*Center for Biophysics and Quantitative Biology, University of Illinois at Urbana-Champaign, Urbana, IL, 61801, United States*

§*Cancer Center at Illinois, University of Illinois at Urbana-Champaign, Urbana, IL, 61801, United States*

E-mail: diwakar@illinois.edu

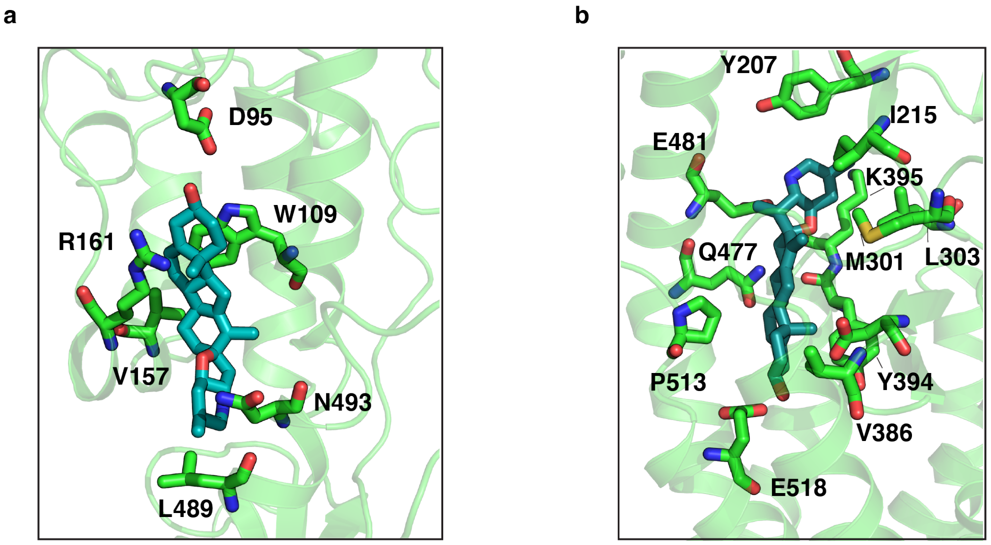

Figure S1: Comparison of the binding sites of cyclopamine. (a) Cyclopamine in SMO-CRD. Cyclopamine forms contacts with 7 residues. (b) Cyclopamine in TMD. Cyclopamine forms contacts with 11 residues.

Table S1: Modelled residues for inactive and active SMO.

Modelled Residues	Constraints	Location
I429	None	ICL3
K430	None	ICL3
S431	None	ICL3
N432	None	ICL3
H433	None	ICL3
P434	None	ICL3
G435	None	ICL3
L436	None	ICL3
L437	None	ICL3
S438	None	ICL3
E439	None	ICL3
K440	α-helical	TM6
A441	α-helical	TM6
A442	α-helical	TM6
S443	α-helical	TM6
K444	α-helical	TM6
I445	α-helical	TM6

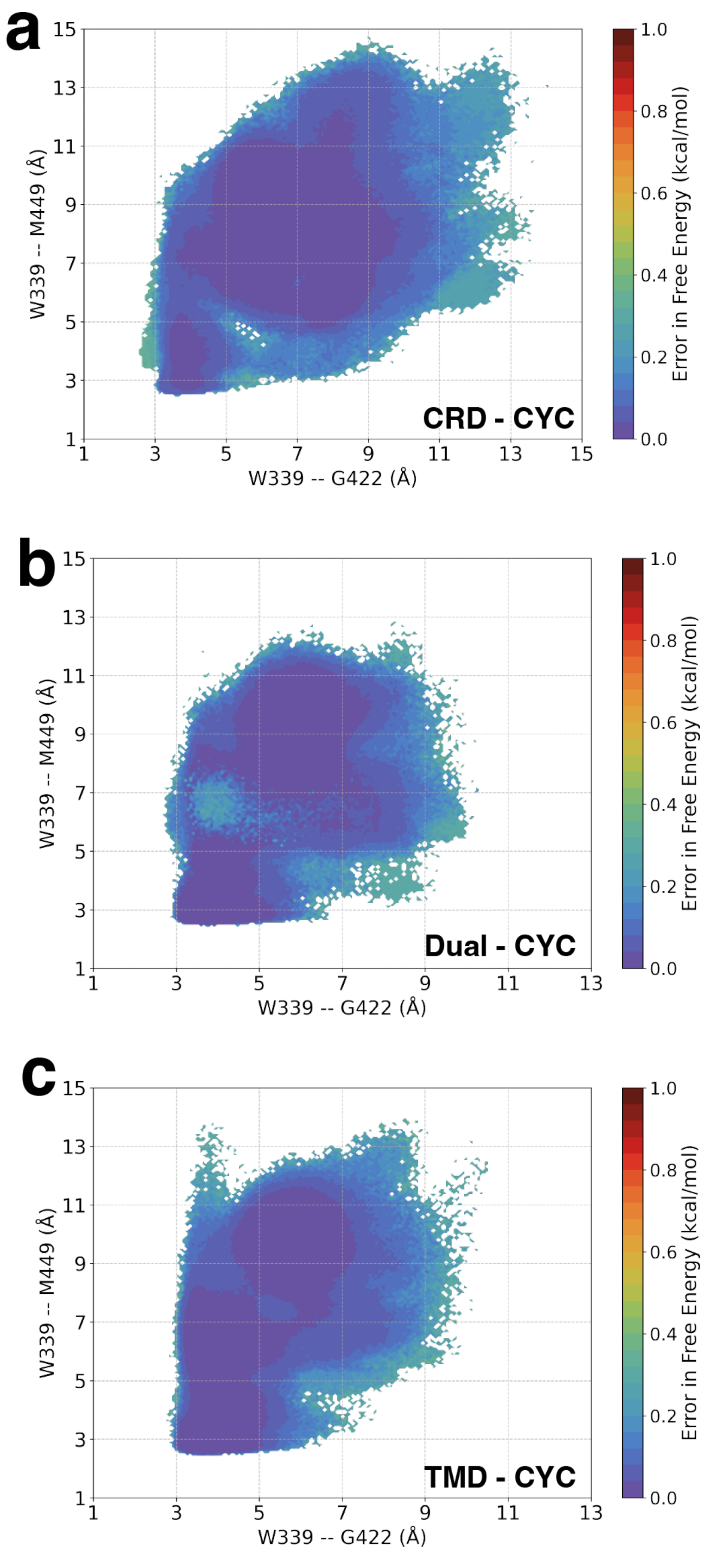

Figure S2: Errors in Figure 2 were calculated by using bootstrapping. 80% of the data was used in 200 iterations.

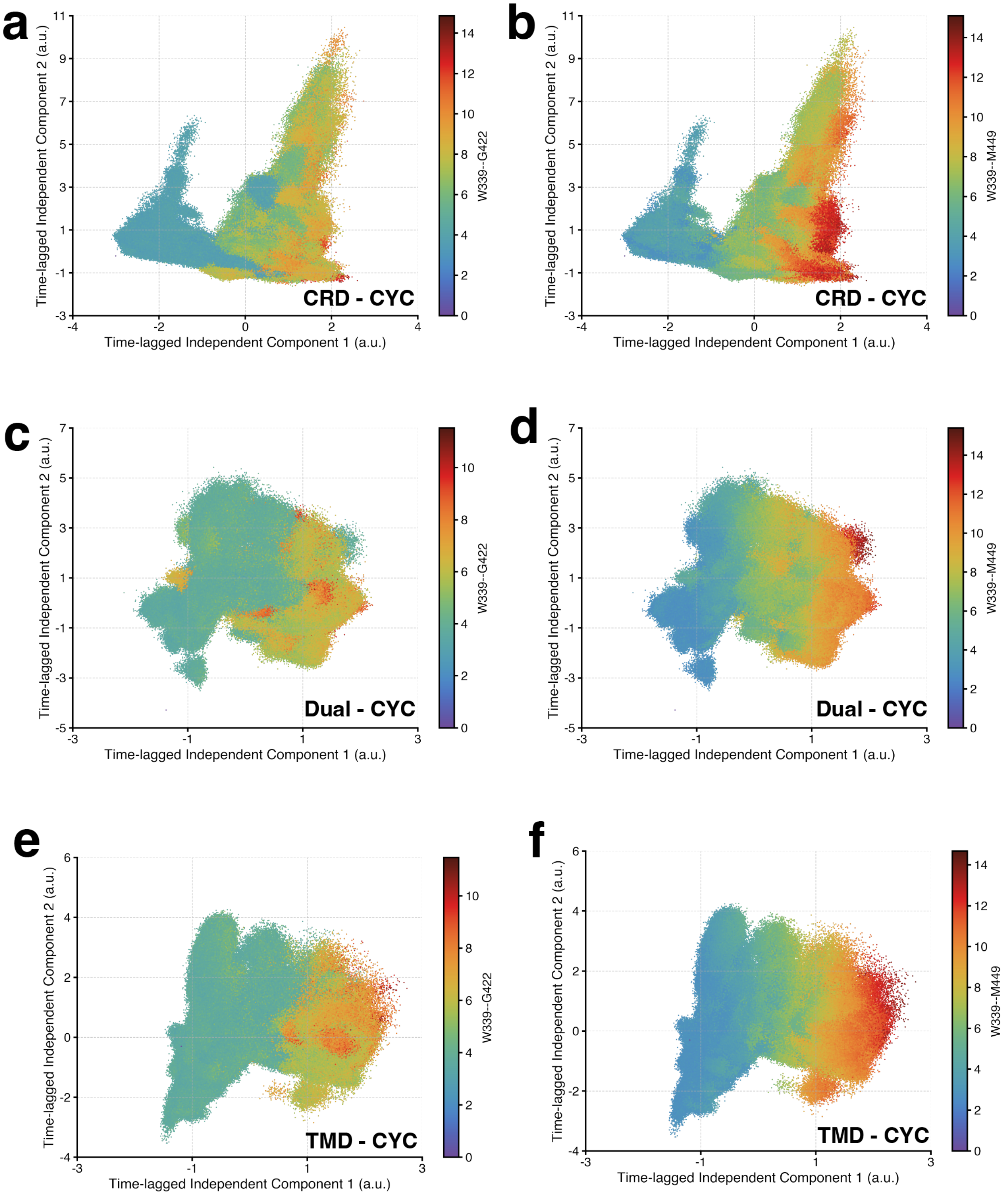

Figure S3: Projection of intracellular residue distances (W339$^{3.50f}$ - G422$^{5.65f}$, W339$^{3.50f}$ - M449$^{6.30f}$) onto the tICA space of (a,b) CRD-CYC, (c,d) Dual-CYC, and (e,f) TMD-CYC. In all cases, tIC1 is related with activation. This demonstrates that all systems can undergo activation through intracellular rearrangement of these residues.

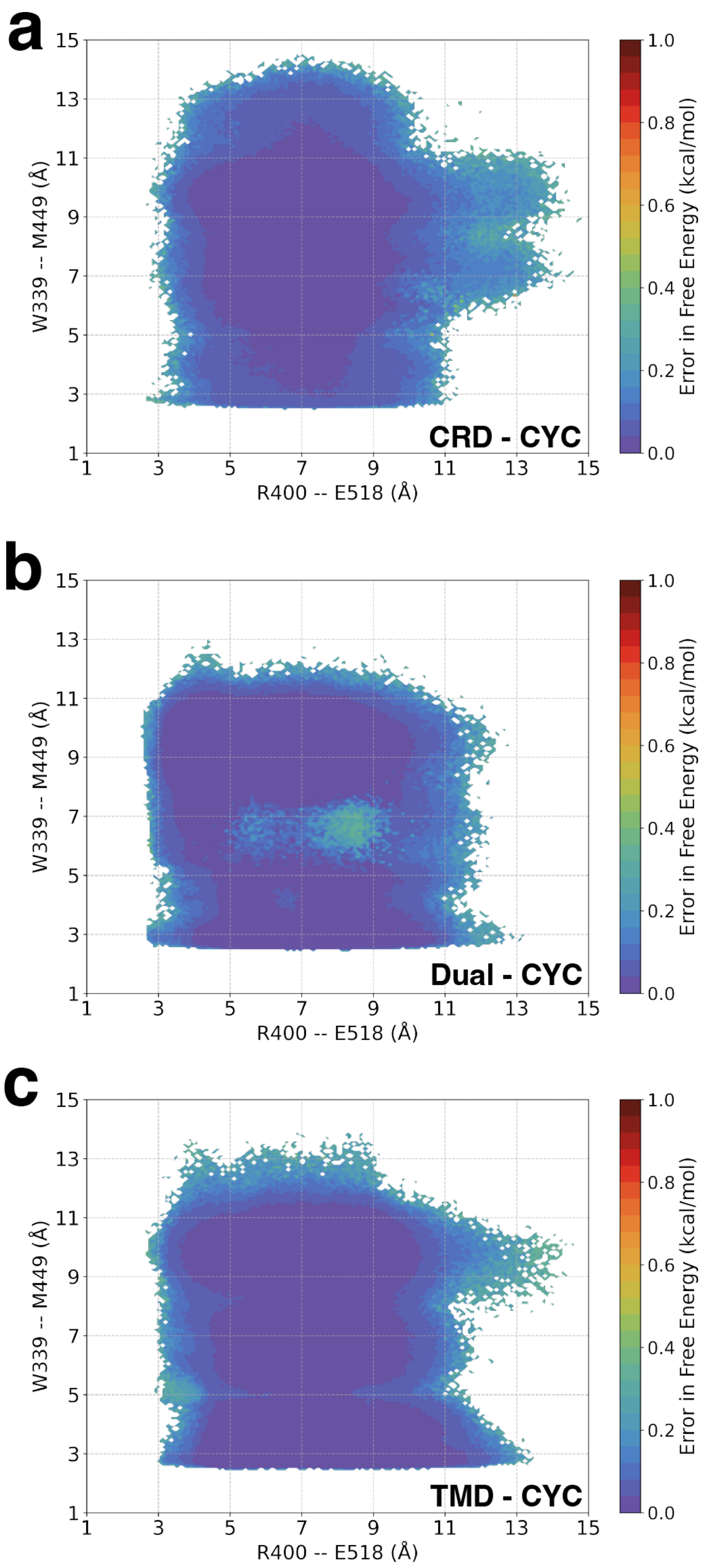

Figure S4: Errors in Figure 3 were calculated by using bootstrapping. 80% of the data was used in 200 iterations.

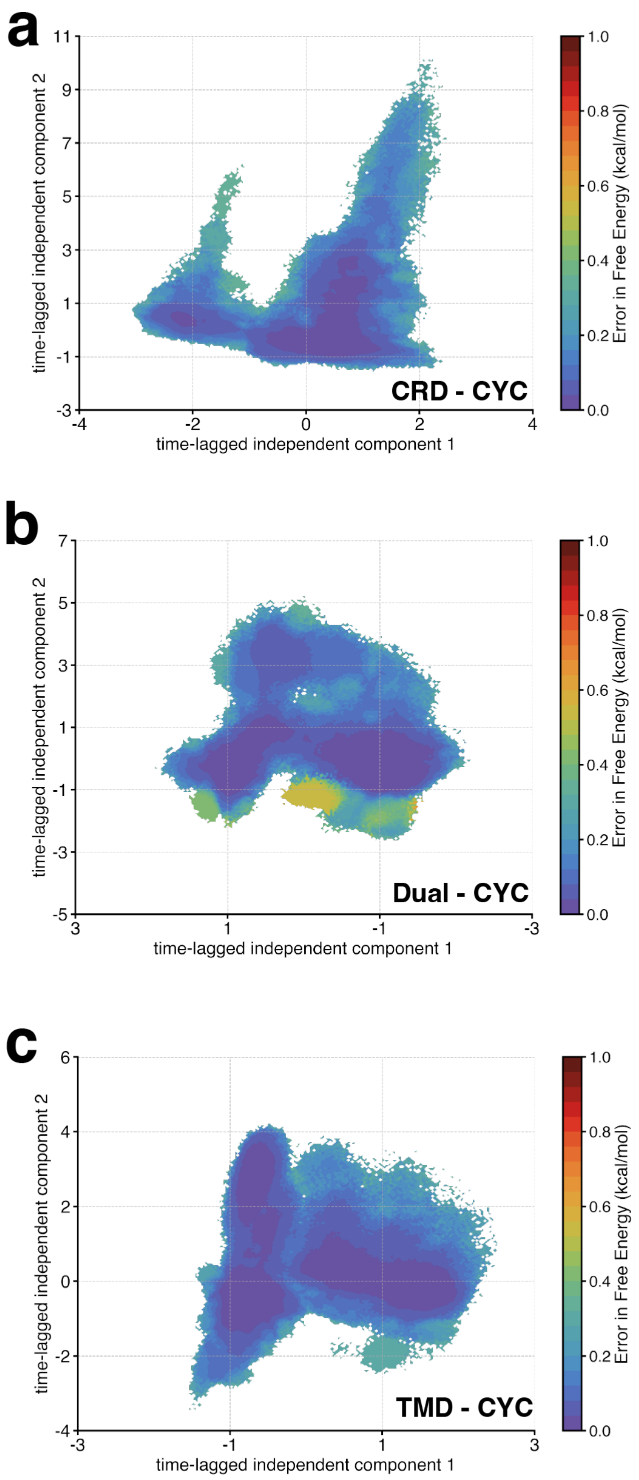

Figure S5: Errors in tICA plots in Figure 4 were calculated by using bootstrapping. 80% of the data was used in 200 iterations.

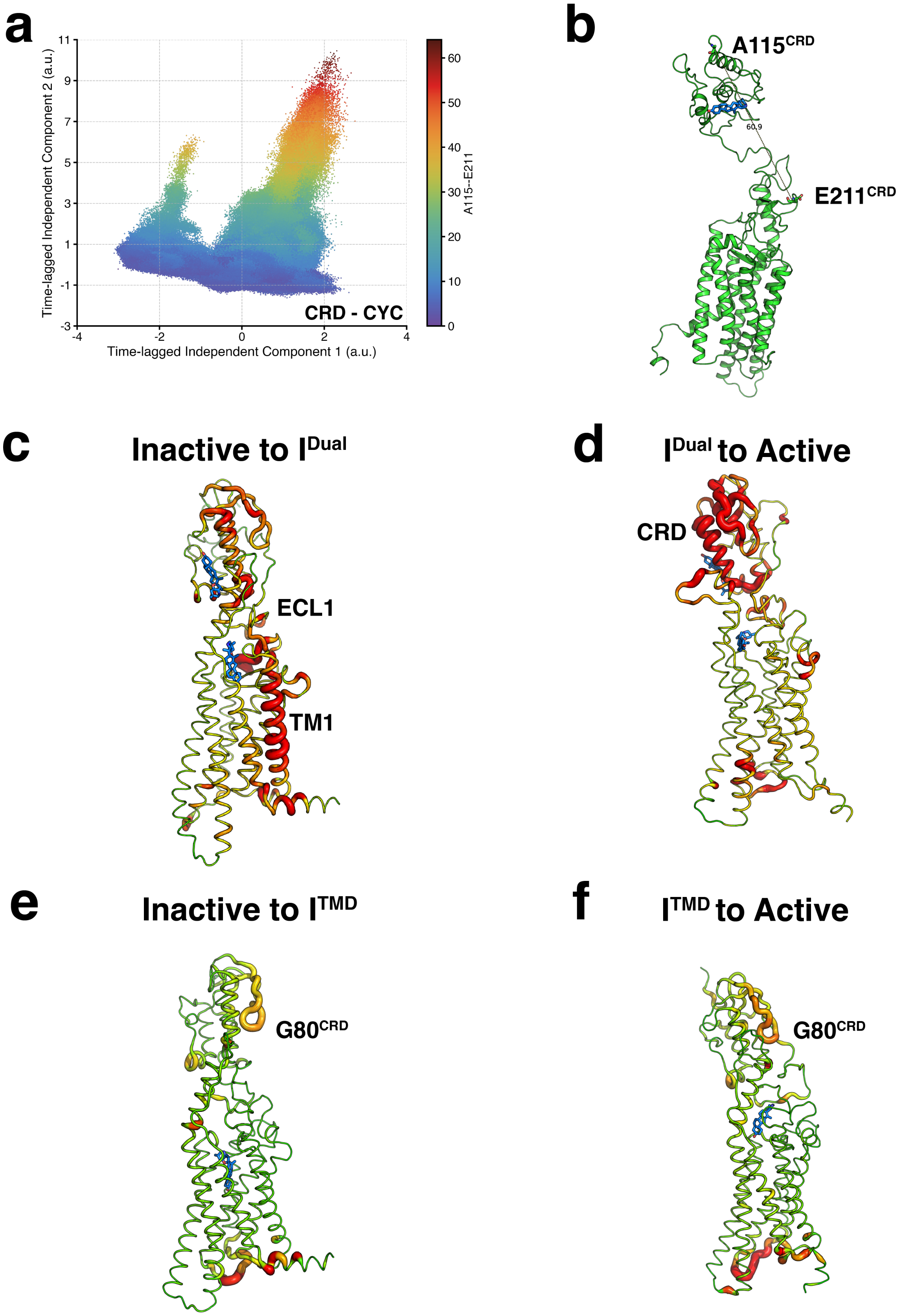

Figure S6: Projection of (a) $A115^{CRD}$ - $E211^{CRD}$ data onto the tICA space of CRD-CYC, (b) Obtained frame of CRD-CYC that has the highest tIC2 value of $A115^{CRD}$ - $E211^{CRD}$, (c) Transition state of Dual-CYC from inactive to I^{Dual} - ECL1 and TM1 are major contributors. (d) Transition state of Dual-CYC from I^{Dual} to active state - CRD is the major contributor. (e) Transition state of TMD-CYC from inactive to I^{TMD} - $G80^{CRD}$ is the major contributor. (f) Transition state of TMD-CYC from I^{TMD} to active state - $G80^{CRD}$ is the major contributor.

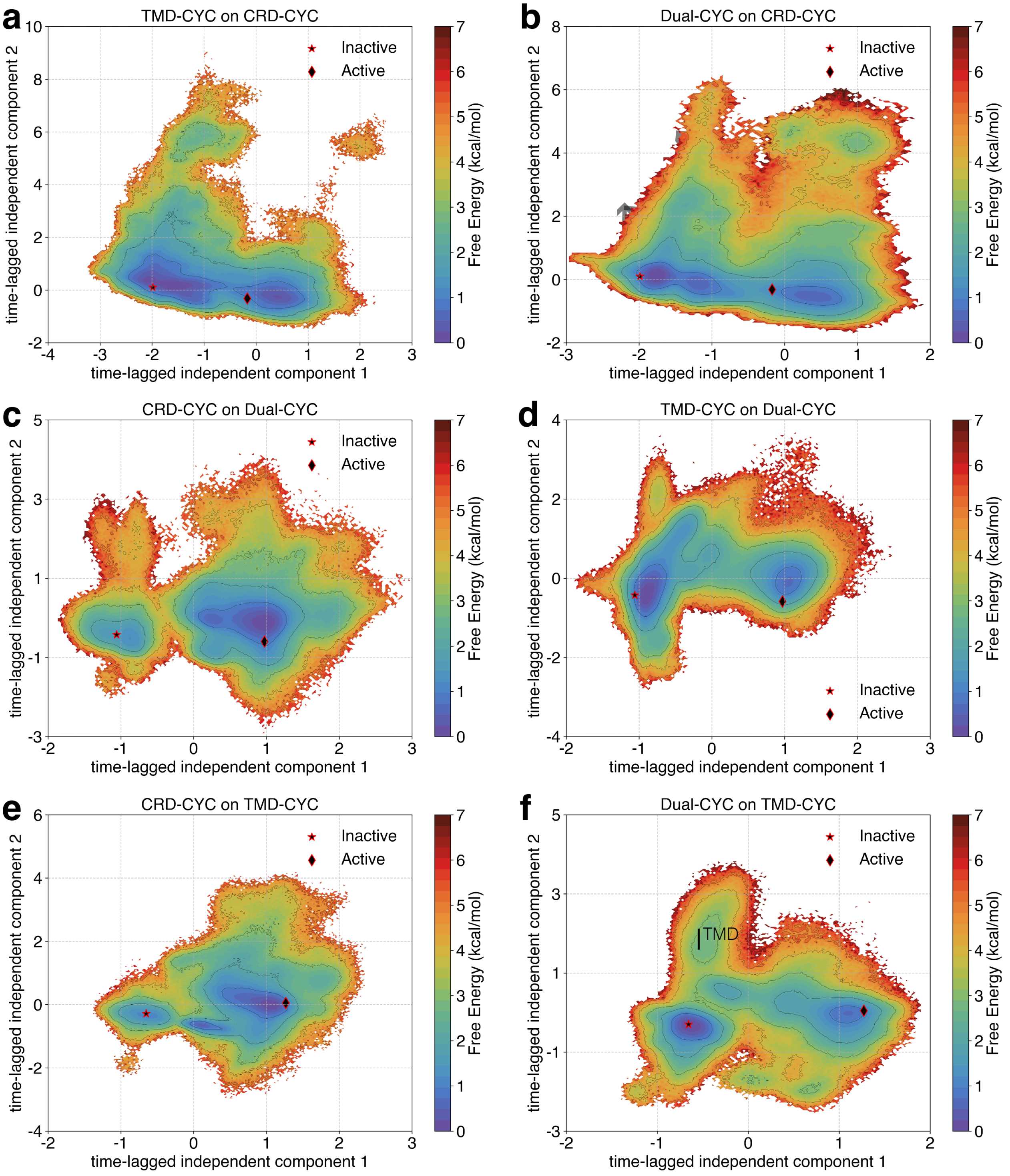

Figure S7: Projection of (a) TMD-CYC data onto the tICA space of CRD-CYC, (b) Dual-CYC data onto CRD-CYC, (c) CRD-CYC data onto Dual-CYC, (d) TMD-CYC data onto Dual-CYC, (e) CRD-CYC data onto TMD-CYC, (f) Dual-CYC data onto TMD-CYC. Figure (f) shows the presence of the intermediate state of TMD-CYC whenever TMD is occupied with cyclopamine. All plots are MSM weighted.

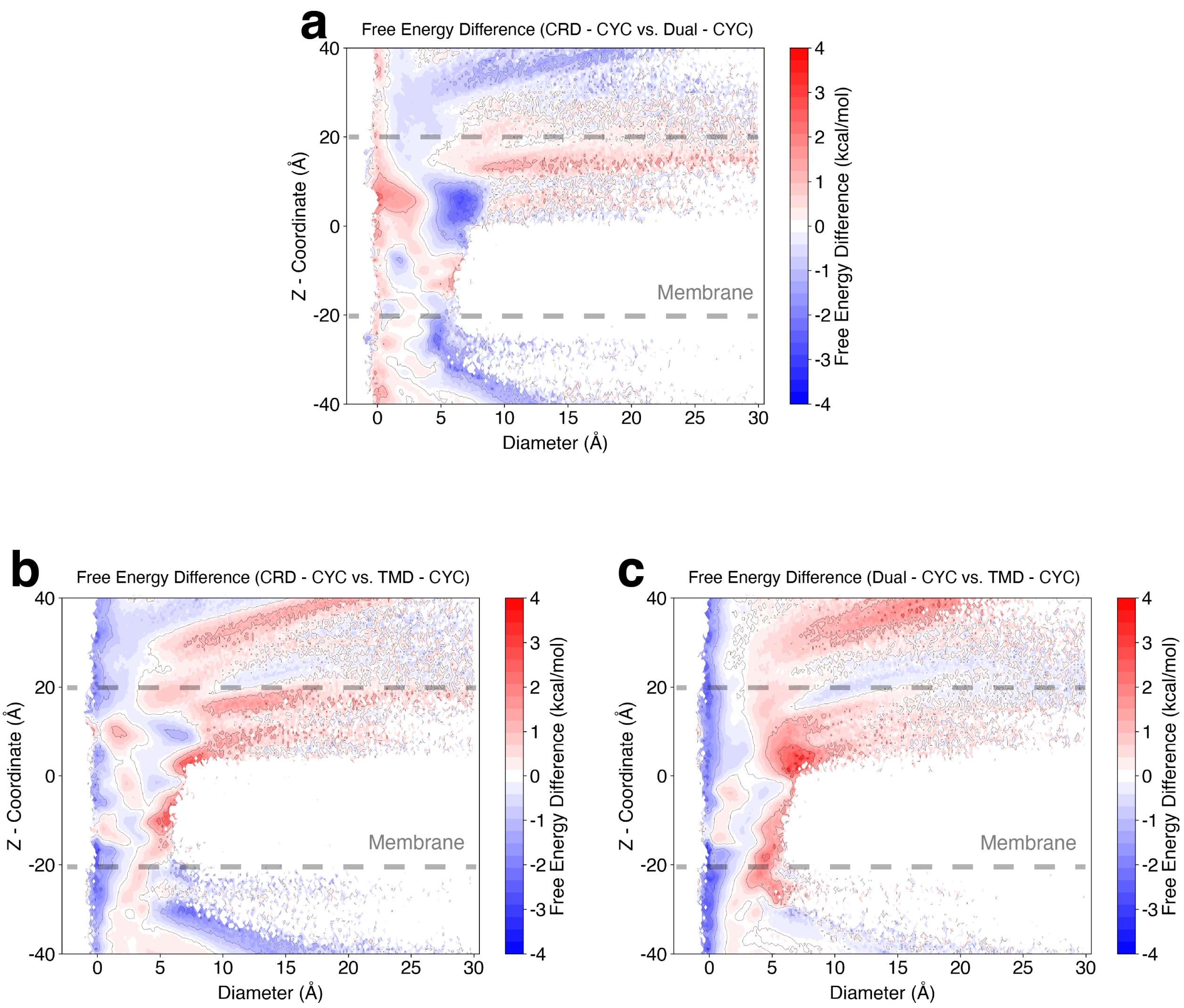

Figure S8: Free energy difference between the three plots shown in Fig 5. Free energy difference between (a) CRD - CYC and Dual - CYC, (b) CRD - CYC and TMD - CYC, (c) Dual - CYC and TMD - CYC. Red color clearly shows the expansion of tunnel for CRD - CYC in the upper leaflet.

Table S2: Membrane composition designed using CHARMM-GUI.

Lipid	Upper Leaflet	Lower Leaflet
Cholesterol	21	21
POPC	76	76
Sphingomyelin	4	4
Total	101	101

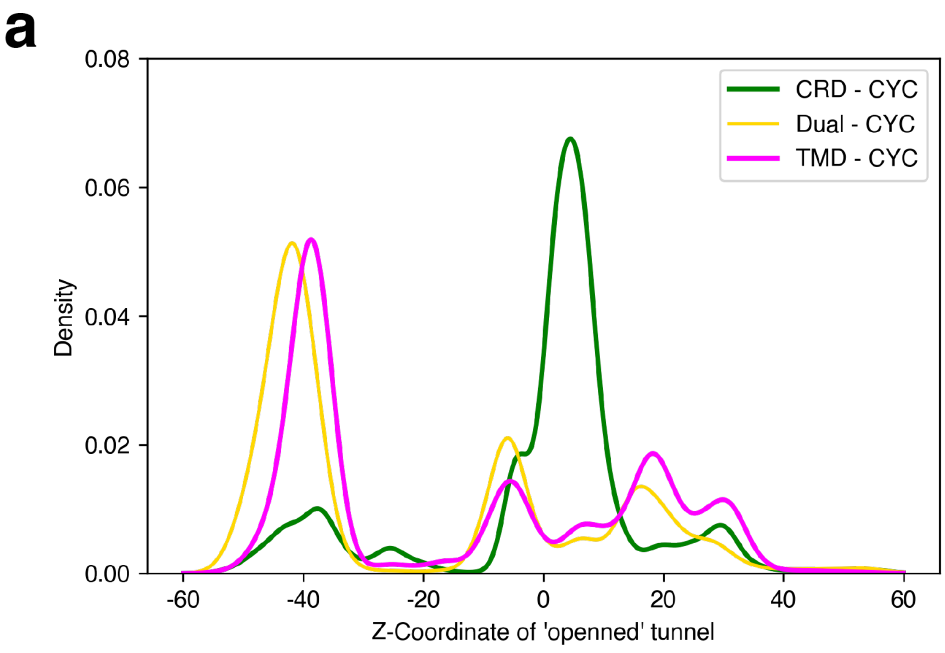

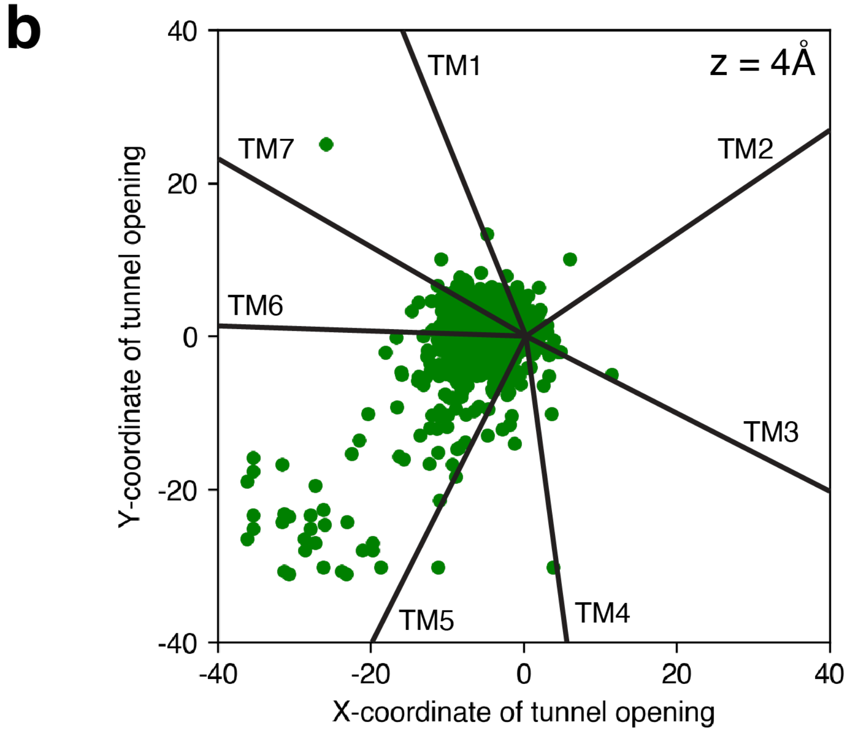

Figure S9: (a) Probability density of Z-coordinate of openned tunnel, where the diameter exceeds 5 angstroms. Simulations show that tunnel opens in the upper leaflet of the membrane at z = 4 Å. (b) The Y vs. X coordinate of the openned tunnel for CRD - CYC. Cluster is centered around the interface of TM5 and TM6. Transmembrane helical boundaries are indicated with black lines.

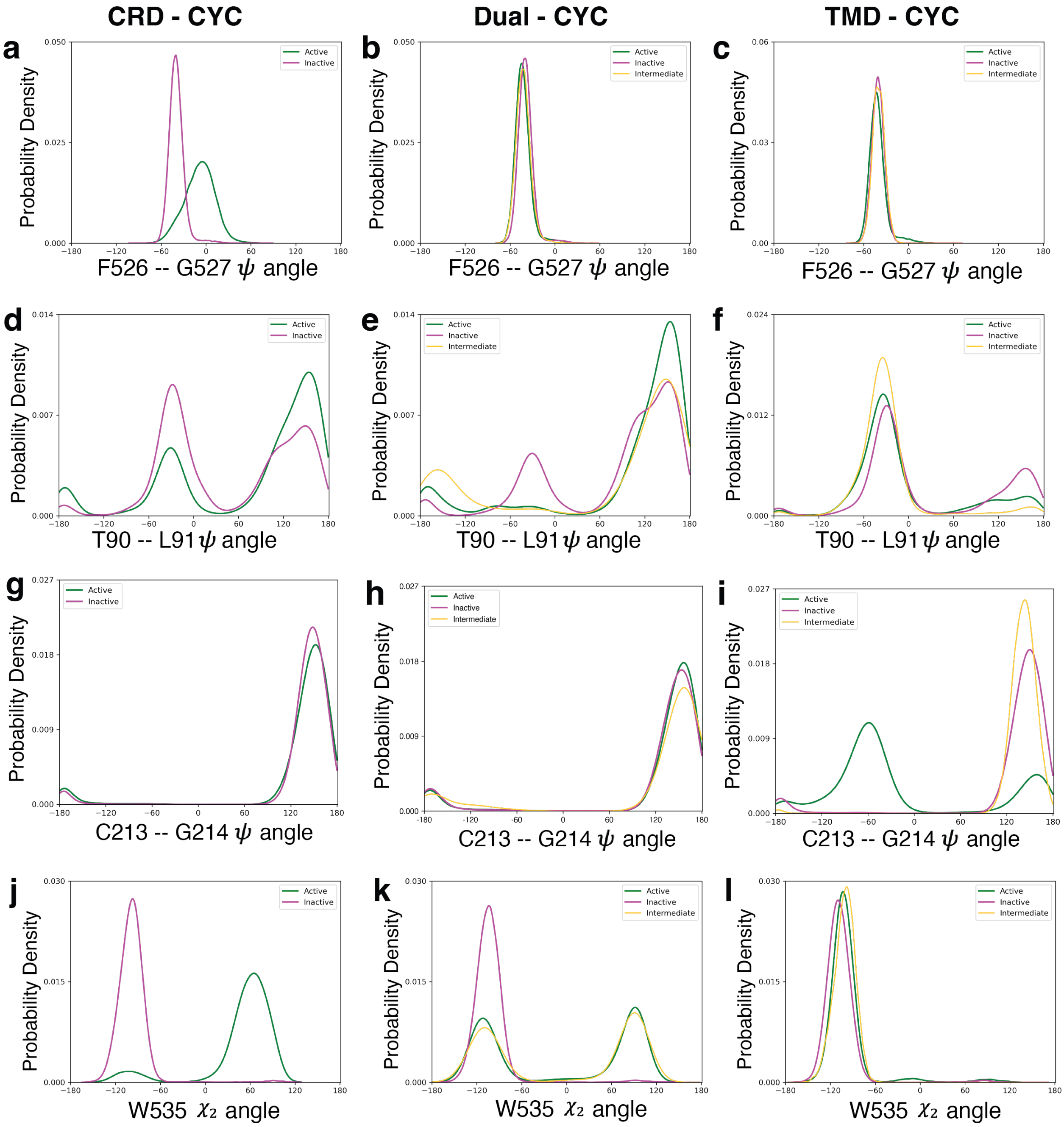

Figure S10: Probability density plots of ψ dihedral angle rotation between F526$^{7.46f}$ and G527$^{7.47f}$ in (a) CRD-CYC, (b) Dual-CYC, (c) TMD-CYC. Probability density plots of ψ dihedral angle rotation between T90^{CRD} and L91^{CRD} in (d) CRD-CYC, (e) Dual-CYC, (f) TMD-CYC. Probability density plots of ψ dihedral angle rotation between C213^{CRD} and G214^{CRD} in (g) CRD-CYC, (h) Dual-CYC, (i) TMD-CYC. Probability density plots of χ_2 dihedral angle rotation at W535$^{7.55f}$ in (j) CRD-CYC, (K) Dual-CYC, (l) TMD-CYC.

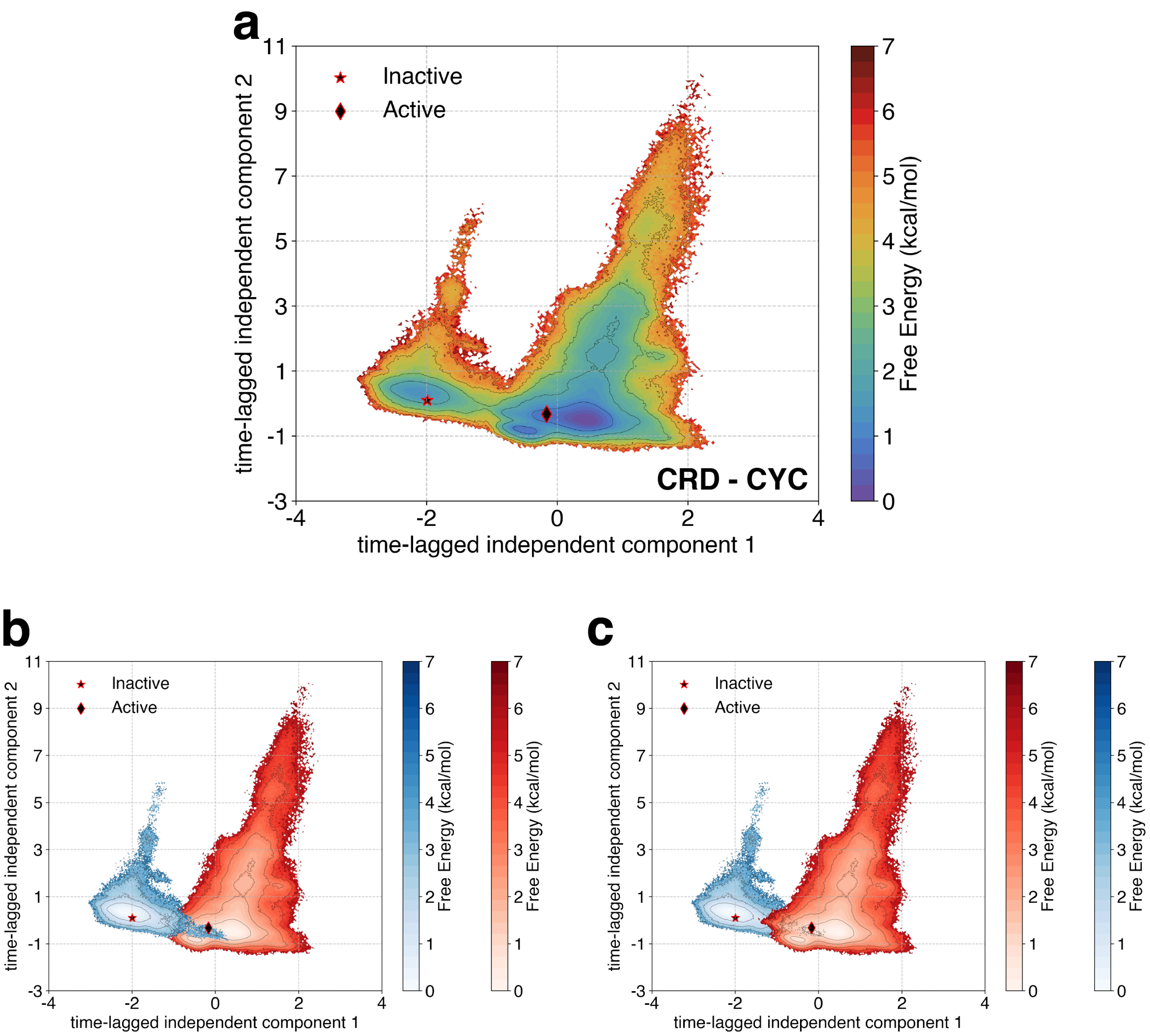

Figure S11: (a) Visualization of CRD - CYC projected onto the the tICA space, utilizing the two slowest components. The inactive and active starting points are marked. (b,c) The data for each inactive and active starting point are projected separately onto the tICA landscape. The overlap of two regions indicate the transitions from inactive to active state. The data from the inactive starting structure is depicted in blue, while the data from the active starting structure is shown in red.

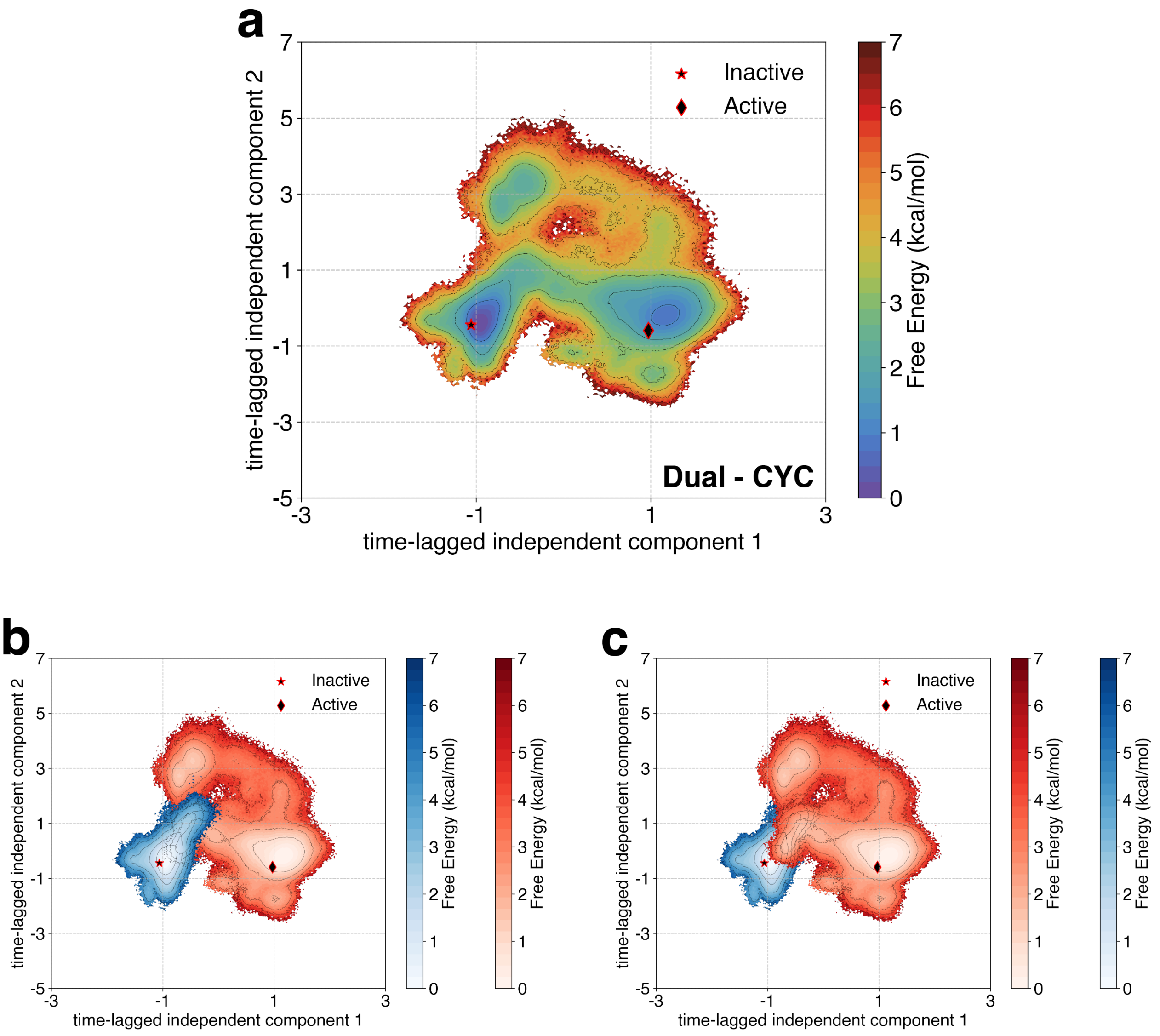

Figure S12: (a) Visualization of Dual - CYC onto the the tICA space, utilizing the two slowest components. The inactive and active starting points are marked. (b,c) The data for each inactive and active starting point are projected separately onto the tICA landscape. The overlap of two regions indicate the transitions from inactive to active state. The data from the inactive starting structure is depicted in blue, while the data from the active starting structure is shown in red.

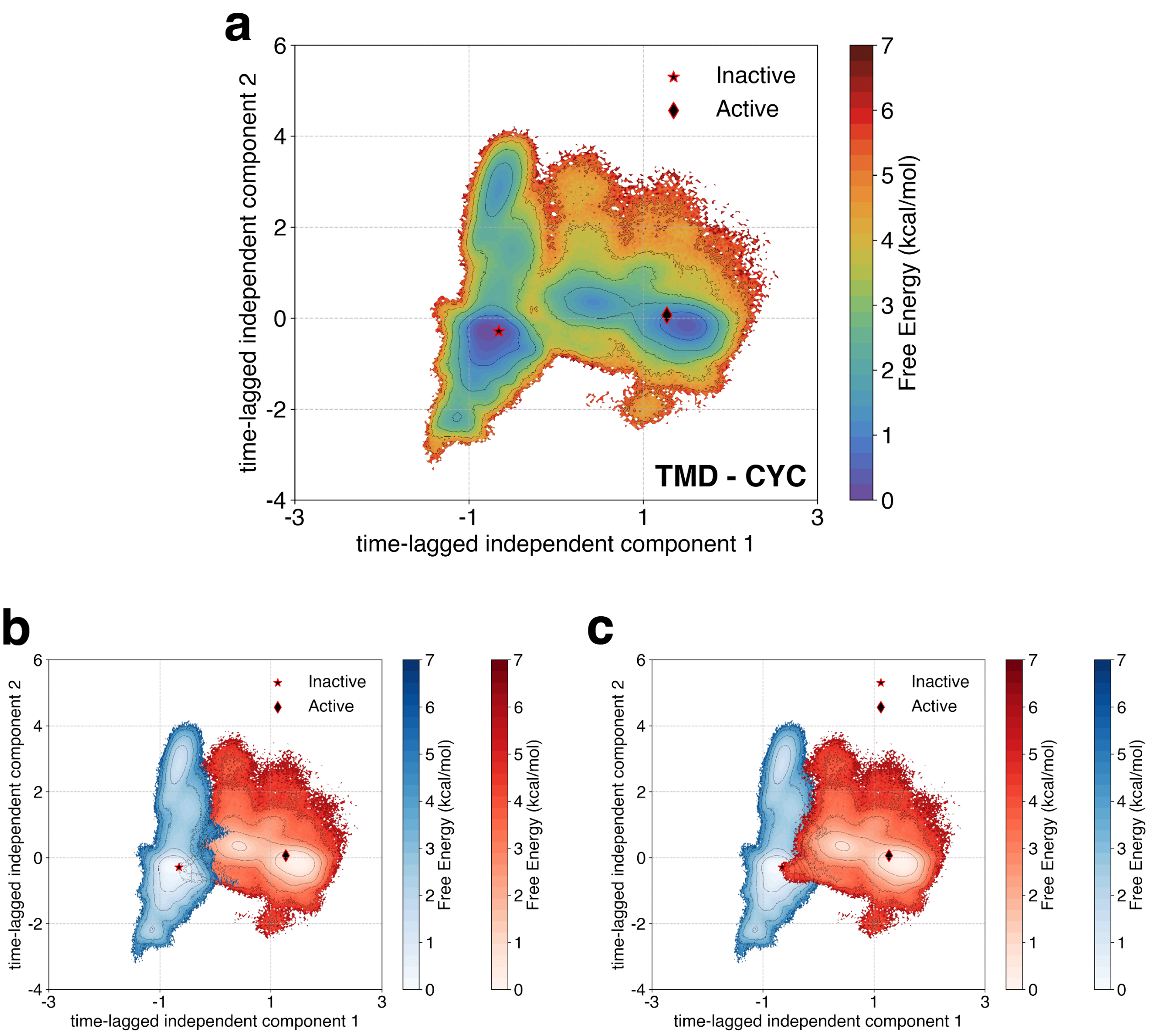

Figure S13: (a) Visualization of TMD - CYC projected onto the the tICA space, utilizing the two slowest components. The inactive and active starting points are marked. (b,c) The data for each inactive and active starting point are projected separately onto the tICA landscape. The overlap of two regions indicate the transitions from inactive to active state. The data from the inactive starting structure is depicted in blue, while the data from the active starting structure is shown in red.

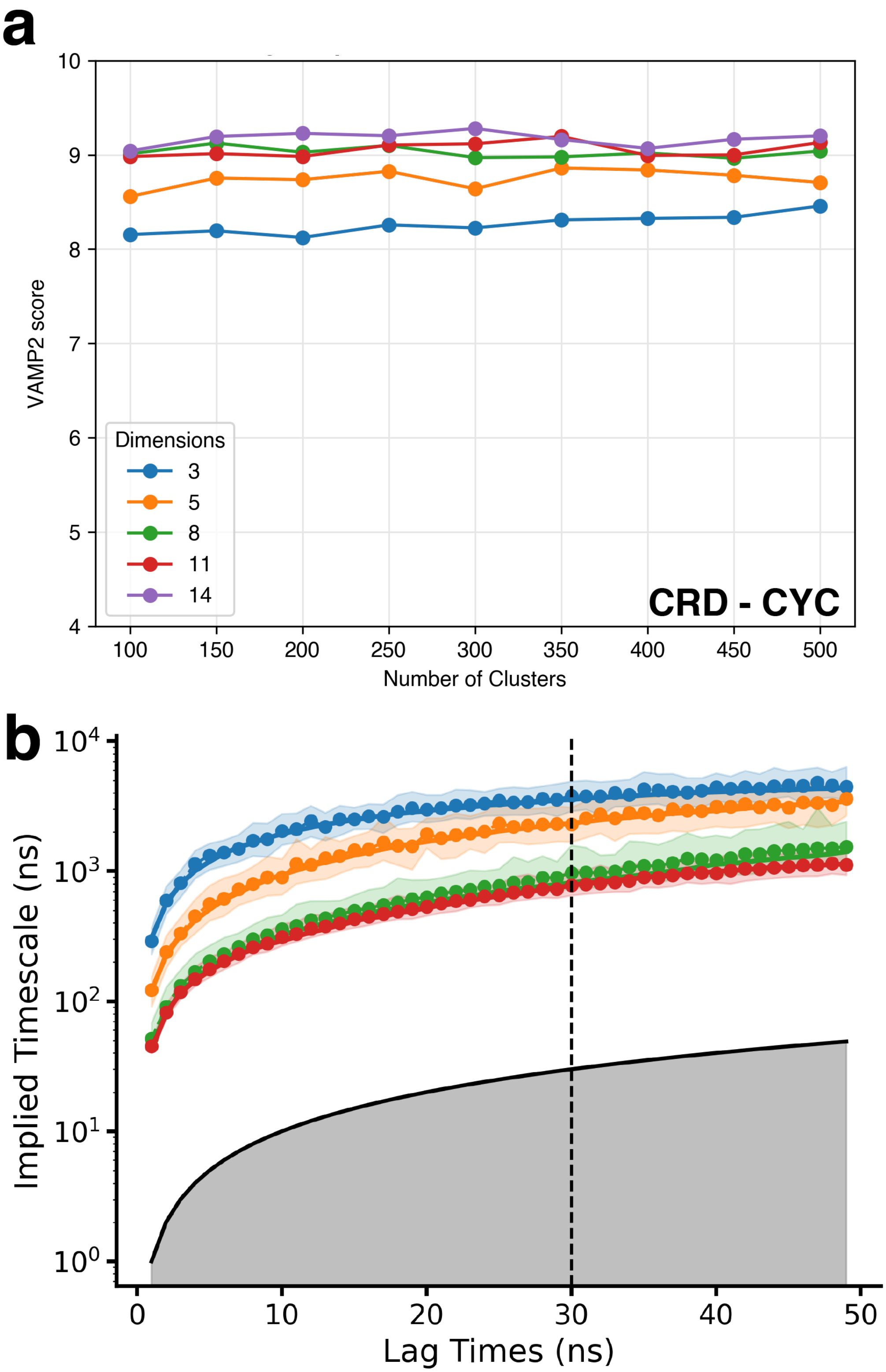

Figure S14: MSM construction for CRD - CYC. (a) VAMP2 score vs. Number of Clusters as a funtion of varying number of tICA components. (b) Implied Timescales vs. Lag Times for MSM with 150 clusters and 11 tICA components. The final MSM lagtime was chosen as 30 ns.

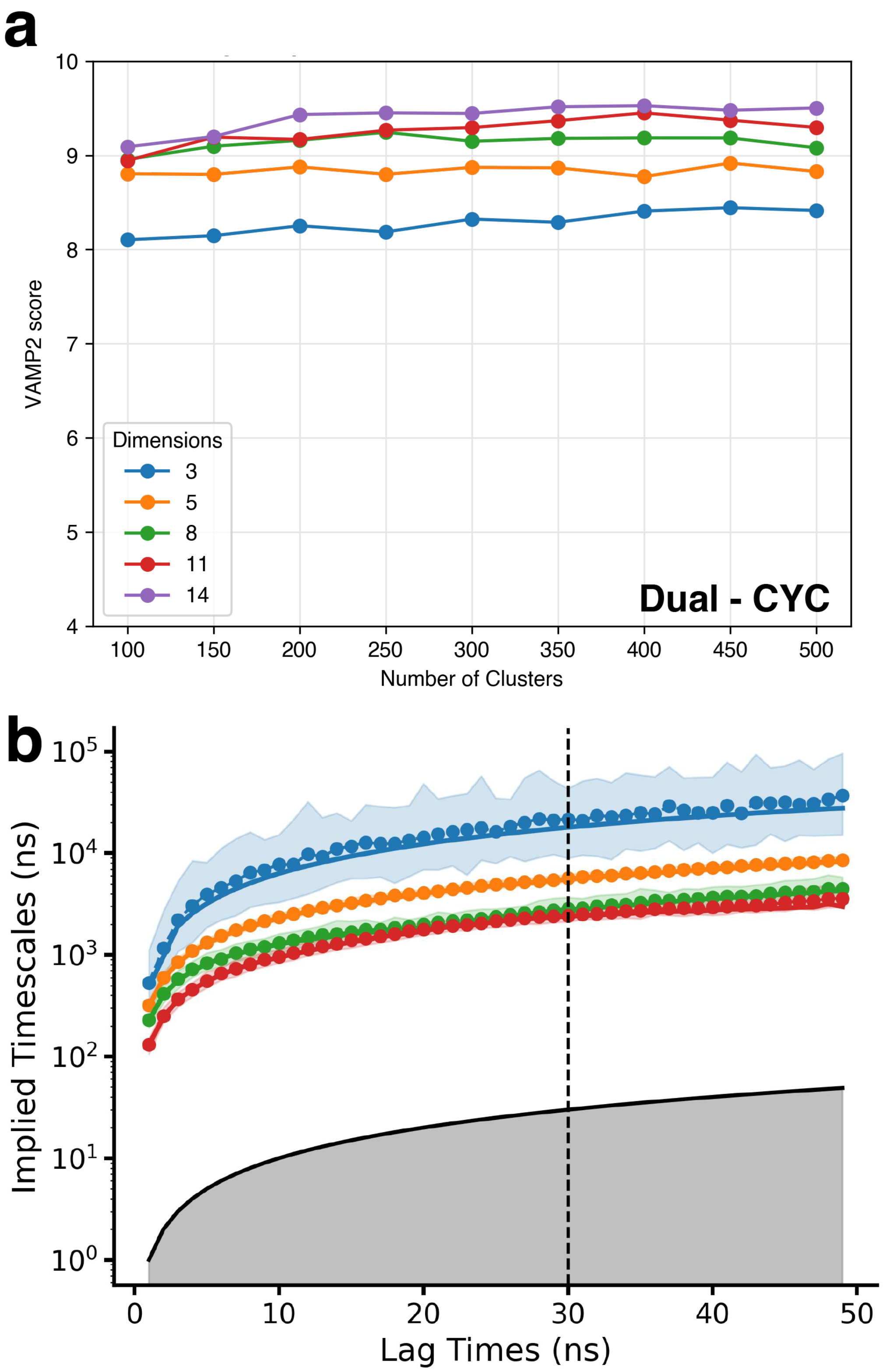

Figure S15: MSM construction for Dual - CYC. (a) VAMP2 score vs. Number of Clusters as a funtion of varying number of tICA components. (b) Implied Timescales vs. Lag Times for MSM with 400 clusters and 14 tICA components. The final MSM lagtime was chosen as 30 ns.

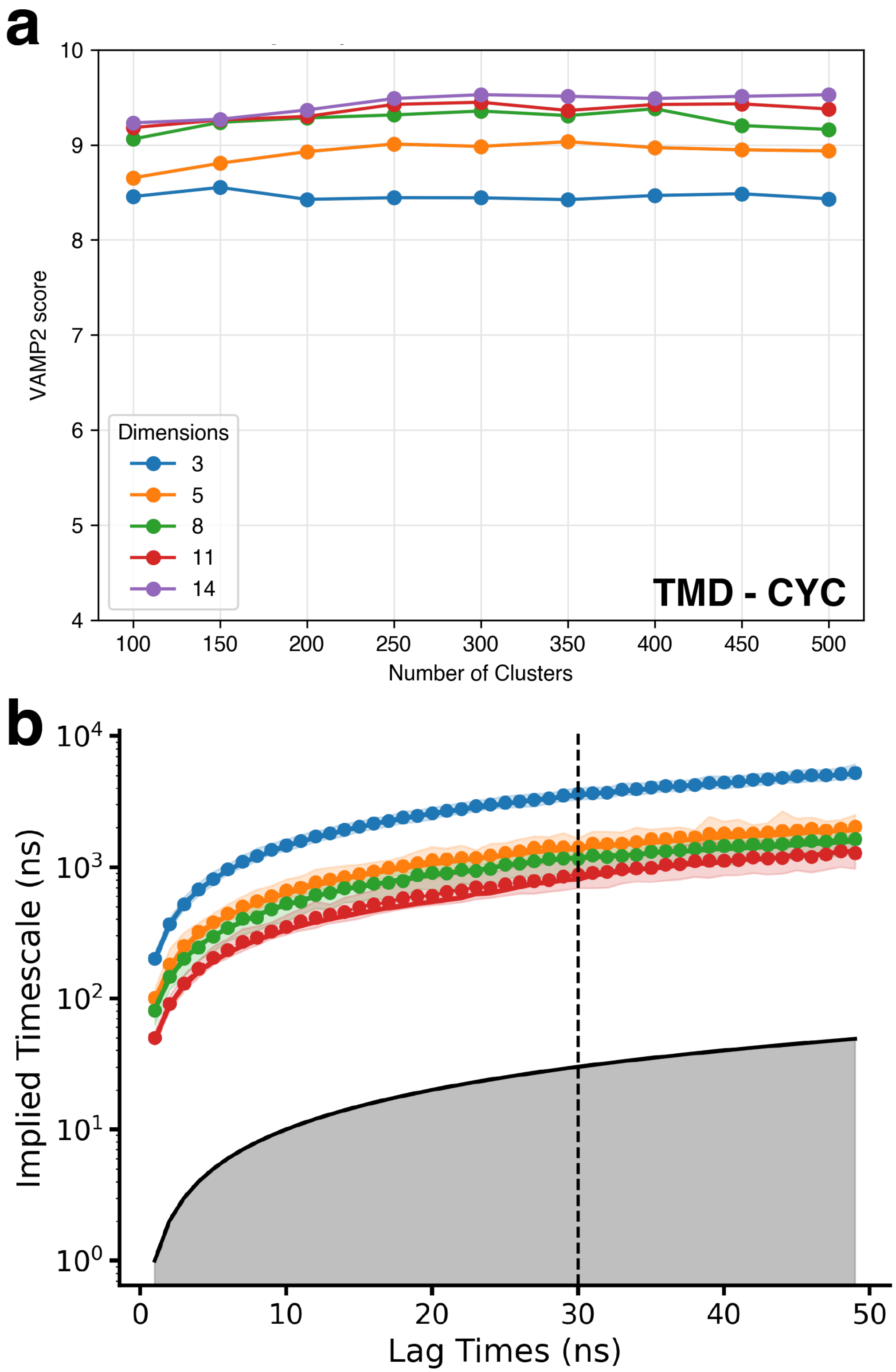

Figure S16: MSM construction for TMD - CYC. (a) VAMP2 score vs. Number of Clusters as a funtion of varying number of tICA components. (b) Implied Timescales vs. Lag Times for MSM with 150 clusters and 11 tICA components. The final MSM lagtime was chosen as 30 ns.

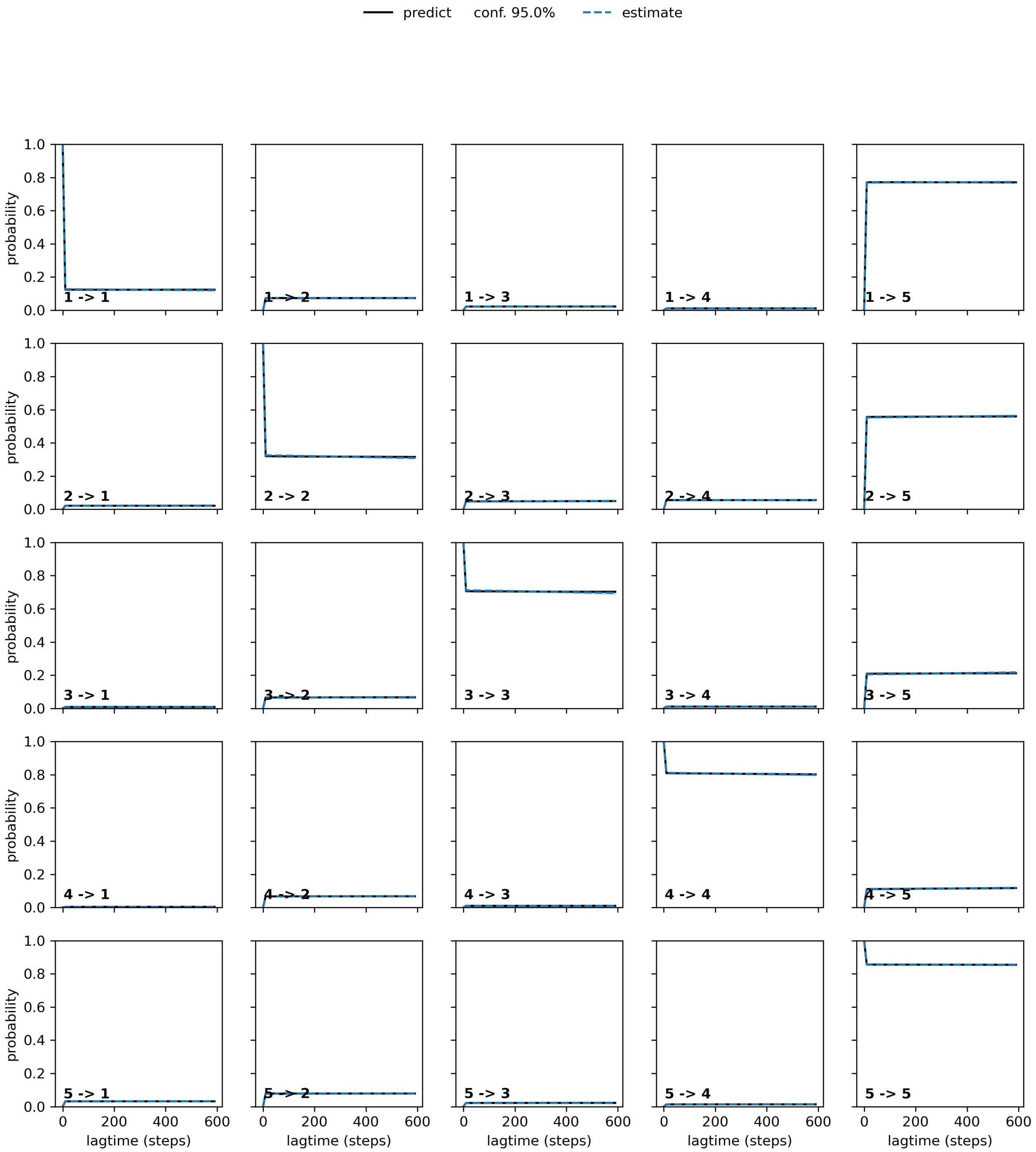

Figure S17: Chapman-Kolmogorov Test performed on CRD - CYC. The test is performed to validate the MSM with 150 clusters and 11 tICA components.

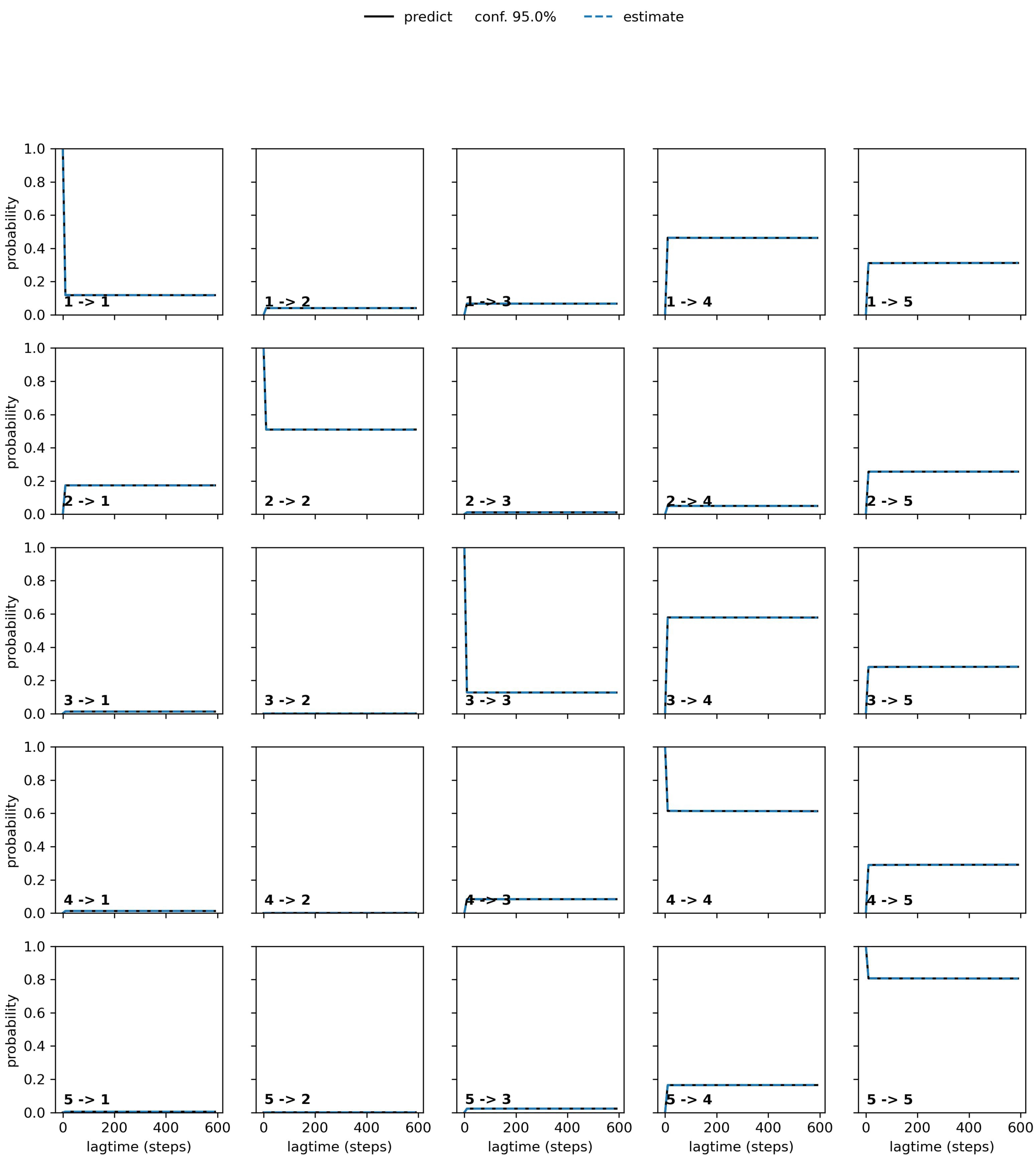

Figure S18: Chapman-Kolmogorov Test performed on Dual - CYC. The test is performed to validate the MSM with 400 clusters and 14 tICA components.

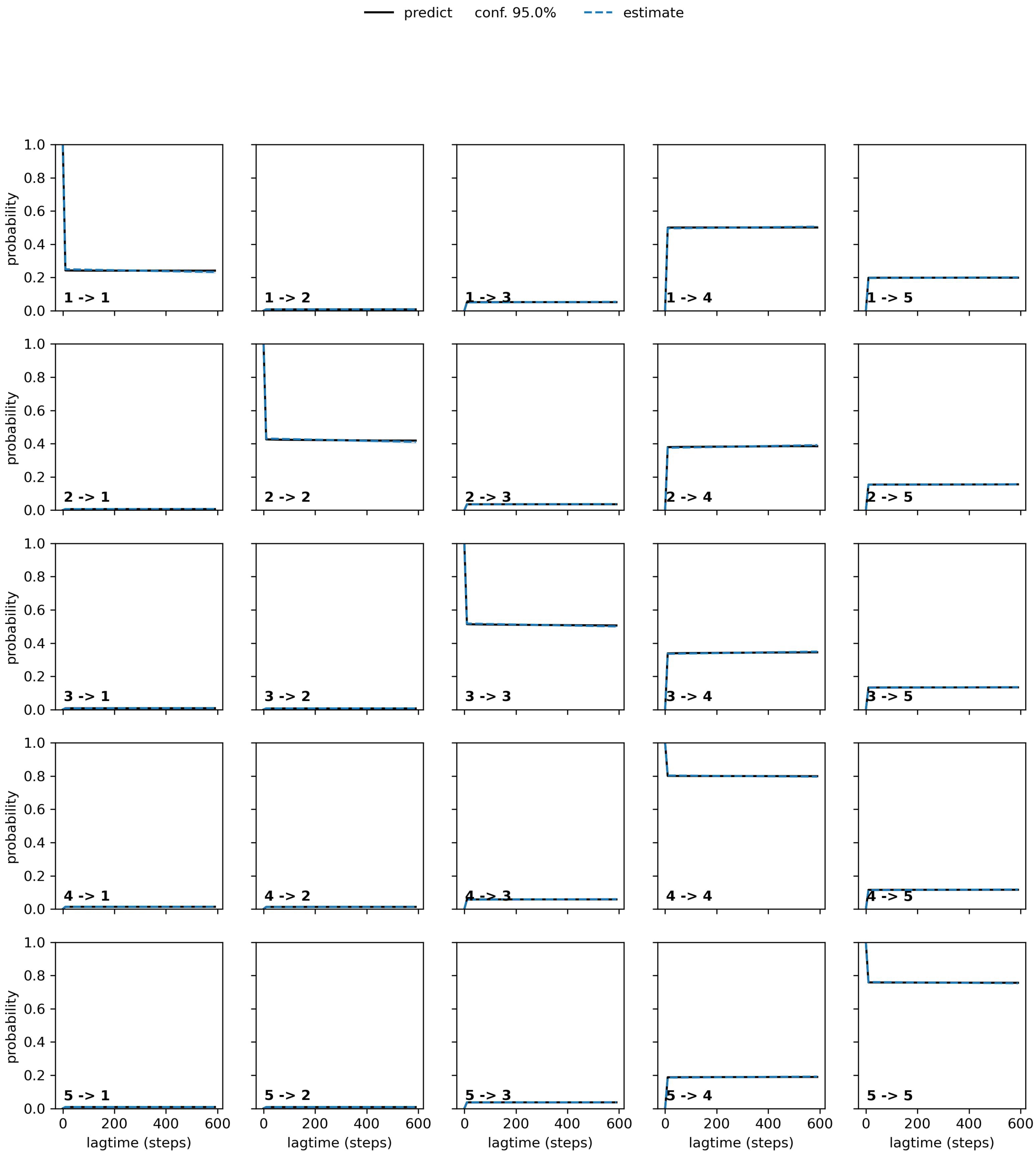

Figure S19: Chapman-Kolmogorov Test performed on TMD - CYC. The test is performed to validate the MSM with 150 clusters and 11 tICA components.

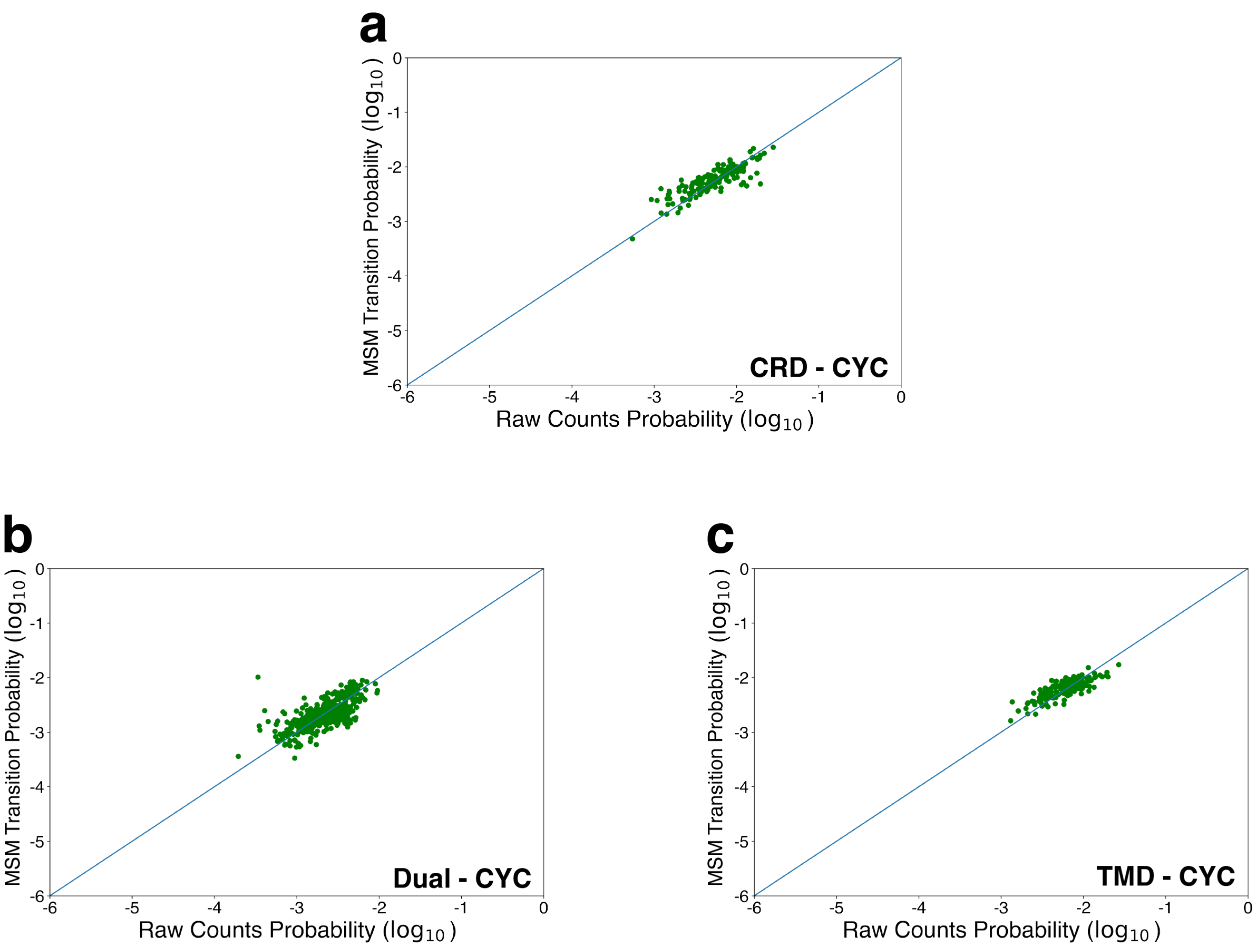

Figure S20: Raw counts versus MSM population for (a) CRD - CYC, (b) Dual - CYC, (c) TMD - CYC.

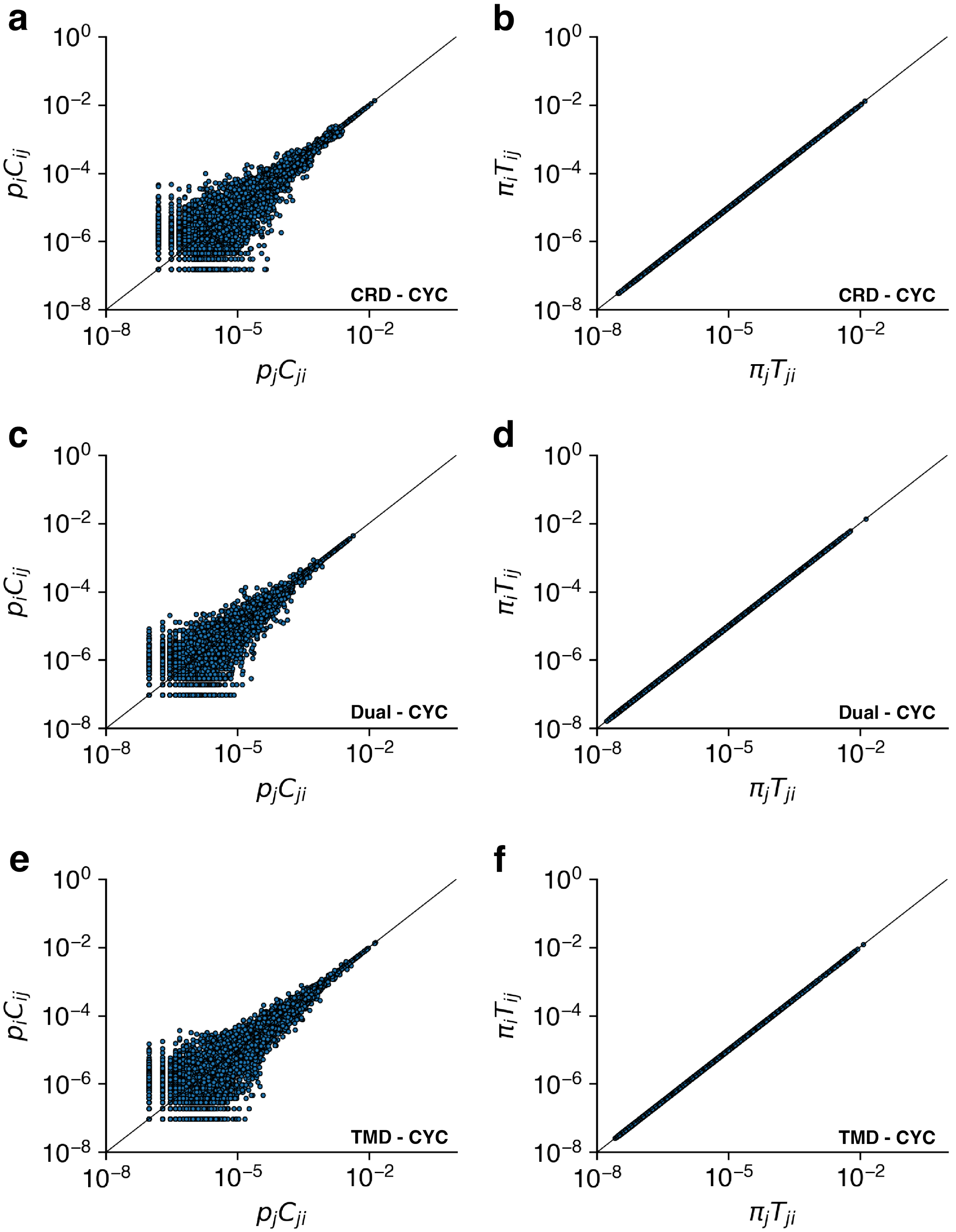

Figure S21: Plots of p_iC_{ij} vs. p_jC_{ji} and π_iT_{ij} vs. π_jT_{ji} for CRD-CYC (a,b), Dual-CYC (c,d), and TMD-CYC (e,f). p_i is the population of state i from raw simulation, π_i is the population of state i from Markov State Model, C_{ij} is the normalized transition counts from state i to state j from count matrix $\mathbf{C}$, and T_{ij} is the transition probability from state i to state j from transition matrix $\mathbf{T}$. The plots show that the transition probabilities are balanced, and the alignment of the points along the diagonal line $y = x$ indicates that the MSMs follow the detailed balance.

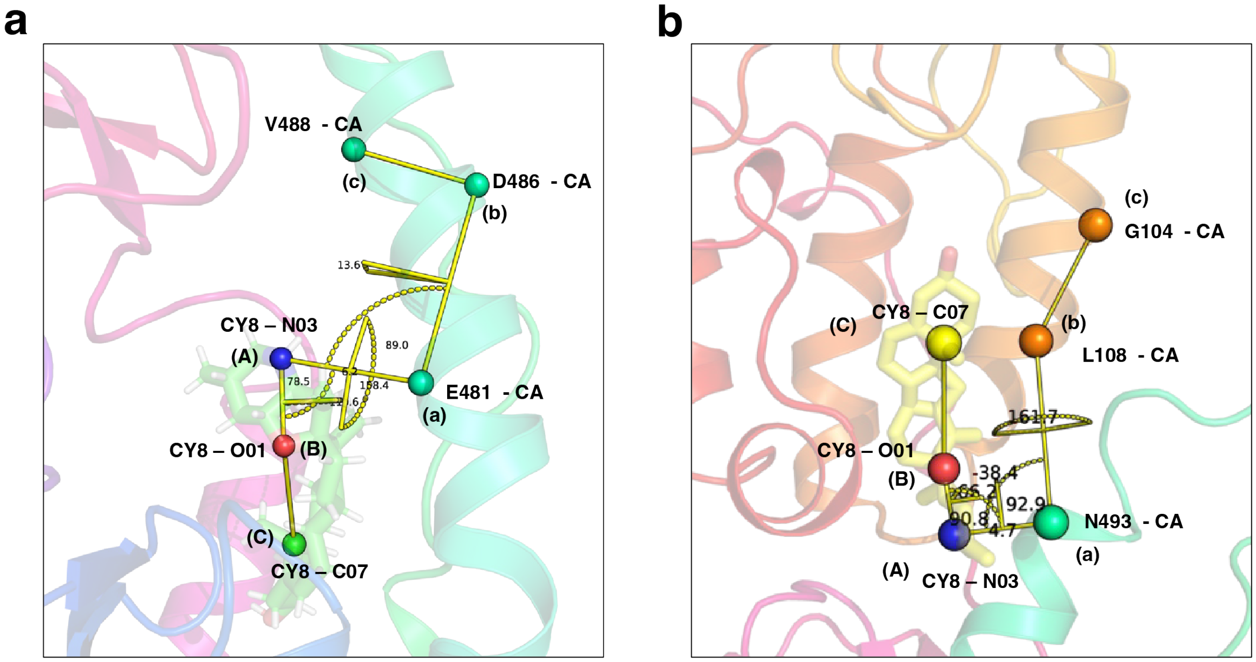

Figure S22: Boresch restraints applied during alchemical calculations for binding free energy computation. (a) Boresch restraints for Cyclopamine bound to TMD of SMO. (b) same as (a), but for Cyclopamine bound to CRD of SMO.

Table S3: 57 pairs of distances used for adaptive sampling. Double lines indicate separation of pairs of residues.

P58	P59	R66	R173	Y85	K133
L106	Y130	L108	W109	W109	L126
R113	W119	A115	E211	R117	W206
R117	G212	W119	Q123	Q123	F187
R151	W163	W163	R168	C169	F174
N202	W206	W206	E211	W206	G212
Y207	E208	Y207	D209	F222	L515
H231	F285	H231	R290	L246	F275
F252	S259	F252	F268	W256	F268
Y262	L353	Q284	R290	G288	E292
R290	R291	F332	A459	L335	W339
W339	G422	W339	M449	W339	G453
W339	W535	F343	M449	L346	I445
L353	F360	K356	F360	Q380	Y399
V381	V392	Y397	F474	Y397	Q477
L419	F457	H433	P434	H433	G435
G435	L436	S443	N446	H470	F474
W480	P513	Y487	Q491	Y487	I509
Q502	I504	L516	K519	T534	W537

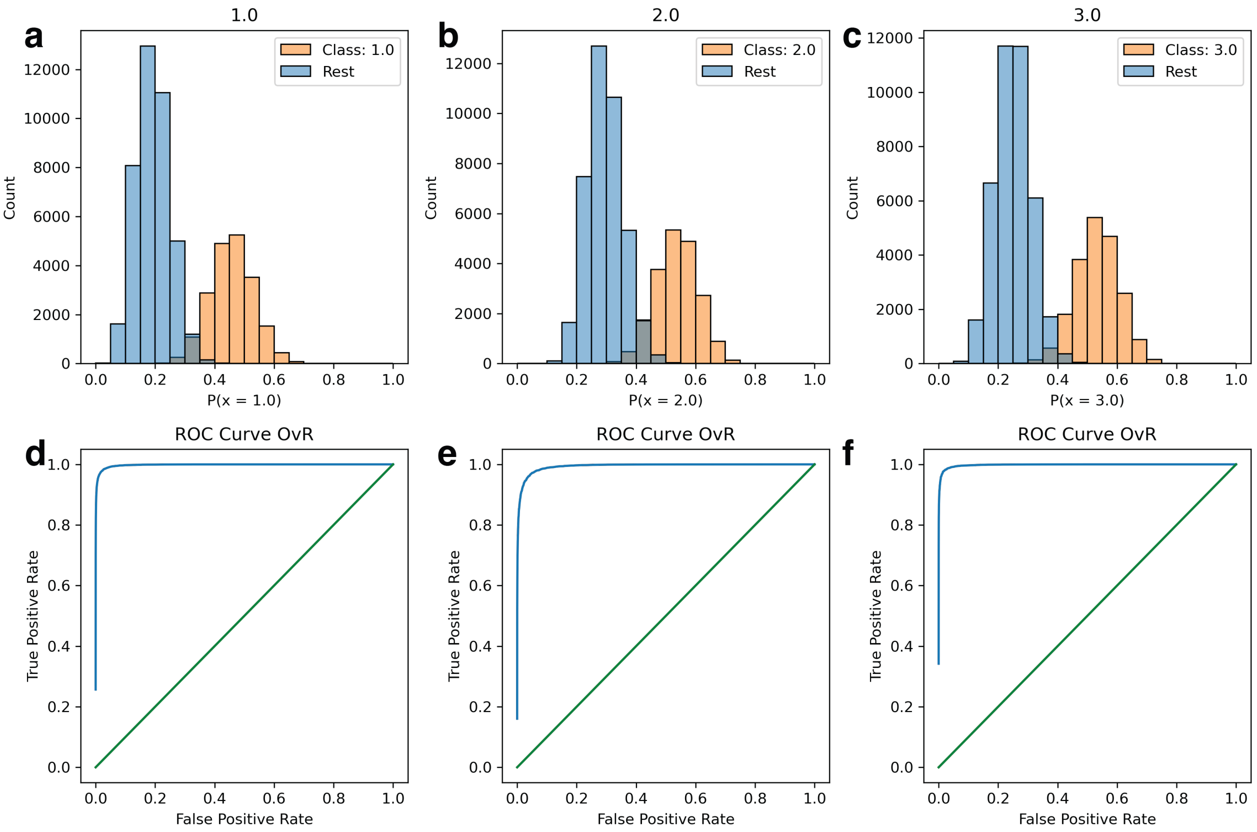

Figure S23: (a)-(c) Class separation Histograms for the 3 classes (a) CRD-CYC (b) Dual-CYC (c) TMD-CYC. The histograms were computed using a one-vs-rest (OvR) strategy. (d)-(f) Characteristic RoC Curves for the multiclass Random Forest Classifier. (d) CRD-CYC, (e) Dual-CYC (f) TMD-CYC. The curves show a classification accuracy greater than 99% for the supplied data.

Table S4: Round wise data collection for CRD - CYC.

Simulation Round	Amount of Data for CRD - CYC
Round 1	200
Round 2	100
Round 3	100
Round 4	100
Round 5	100
Round 6	100
Total	700 µs

Table S5: Round wise data collection for Dual - CYC.

Simulation Round	Amount of Data for Dual - CYC
Round 1	200
Round 2	200
Round 3	200
Round 4	200
Round 5	200
Round 6	150
Total	1150 µs

Table S6: Round wise data collection for TMD - CYC.

Simulation Round	Amount of Data for TMD - CYC
Round 1	200
Round 2	200
Round 3	200
Round 4	200
Round 5	100
Round 6	100
Round 7	100
Total	1100 µs

Table S7: System details for simulations of SMO-CYC systems.

System	Simulation Box Size (Å^3)	Number of atoms	Number of water molecules	Salt Concentration (mM)
TMD-CYC (Inactive)	85.8 x 85.8 x 153.6	106056	24619	150
TMD-CYC (Active)	85.7 x 85.7 x 151.8	104357	24054	150
CRD-CYC (Inactive)	85.7 x 85.7 x 153.5	105894	24565	150
CRD-CYC (Active)	85.8 x 85.8 x 151.8	104552	24119	150
Dual-CYC (Inactive)	85.8 x 85.8 x 153.5	105995	24575	150
Dual-CYC (Active)	85.8 x 85.8 x 151.8	104434	24056	150
